# How Ebola Impacts Genetics of Western Lowland Gorilla Populations

**DOI:** 10.1371/journal.pone.0008375

**Published:** 2009-12-18

**Authors:** Pascaline J. Le Gouar, Dominique Vallet, Laetitia David, Magdalena Bermejo, Sylvain Gatti, Florence Levréro, Eric J. Petit, Nelly Ménard

**Affiliations:** 1 UMR 6553 Ecobio, Université Rennes1/CNRS, Paimpont, France; 2 ECOFAC/Departamento Biología Animal, Universidad de Barcelona, Barcelona, Spain; 3 Laboratoire d'Ecologie et de Neuro-Ethologie Sensorielles - EA 3988, Université Jean Monnet, Saint Etienne, France; 4 UMR1099 BiO3P, INRA/Agrocampus Rennes/Université Rennes 1, Le Rheu, France; Institute of Molecular and Cell Biology, Singapore

## Abstract

**Background:**

Emerging infectious diseases in wildlife are major threats for both human health and biodiversity conservation. Infectious diseases can have serious consequences for the genetic diversity of populations, which could enhance the species' extinction probability. The Ebola epizootic in western and central Africa induced more than 90% mortality in Western lowland gorilla population. Although mortality rates are very high, the impacts of Ebola on genetic diversity of Western lowland gorilla have never been assessed.

**Methodology/Principal Findings:**

We carried out long term studies of three populations of Western lowland gorilla in the Republic of the Congo (Odzala-Kokoua National Park, Lossi gorilla sanctuary both affected by Ebola and Lossi's periphery not affected). Using 17 microsatellite loci, we compared genetic diversity and structure of the populations and estimate their effective size before and after Ebola outbreaks. Despite the effective size decline in both populations, we did not detect loss in genetic diversity after the epizootic. We revealed temporal changes in allele frequencies in the smallest population.

**Conclusions/Significance:**

Immigration and short time elapsed since outbreaks could explain the conservation of genetic diversity after the demographic crash. Temporal changes in allele frequencies could not be explained by genetic drift or random sampling. Immigration from genetically differentiated populations and a non random mortality induced by Ebola, i.e., selective pressure and cost of sociality, are alternative hypotheses. Understanding the influence of Ebola on gorilla genetic dynamics is of paramount importance for human health, primate evolution and conservation biology.

## Introduction

Emerging infectious diseases in wildlife, raising from complex relationships between social and environmental factors, are accepted as a major threat for both human health and biodiversity conservation [1]–[7]. These diseases often reduce abundance of wildlife populations to low density, increasing their extinction probability, especially in the case of frequency-dependent outbreaks [8]–[10]. Although infectious diseases contribute to the extinct and critically endangered UICN red list status for only 12% of listed species [11], devastating effect of diseases on population demography have been reported for various taxa (see review in [12]).

Among emerging diseases, the Ebola virus received a lot of attention from human health and conservation biology scientists because of his rapid spread through central Africa and the high mortality rate it induced in humans and nonhuman primates [13]–[24]. The first outbreaks occurred in Zaire in 1976 and in the ensuing years until nowadays with mortality rates ranging from 50 to 90% in human populations [22]. The anthropogenic habitat changes and bushmeat hunting lead humans and primates into closer and more frequent contacts which increase the risks of Ebola disease transmission [2], [3], [20], [21], [25]. The disease seems to emerge through spillover from reservoir hosts (maybe the bats [26]) and then it is transmitted between apes, with potential inter ape species transmission [27]. The social structure of ape populations enhances the disease propagation [10], with individuals living in groups being more affected than solitary individuals, what has been named the cost of sociality [28]. Outbreaks have been identified yearly from 2001 to 2005 in the Republic of the Congo, with 294 deaths recorded in human populations (http://www.who.int/csr/don/archive/country/cog/en/index.html acceded the 06.17.2009) and thousands of fatalities for non human primates as chimpanzees (*Pan troglodytes troglodytes*) and Western lowland gorilla (*Gorilla gorilla gorilla*) [13], [23], [28]. In 2002–3 and 2003–4, two epizootics affected the Lossi Gorilla Sanctuary and two-thirds of the local population of Western lowland gorillas disappeared [13]. By 2003–4, Ebola outbreaks happened 50 km far away from the Lossi Sanctuary in the Odzala-Kokoua National Park, which initially homed 20,000 gorillas [28], [29]. The catastrophic demographic decline mainly due to Ebola disease led the World Conservation Union (IUCN) to move the Western lowland gorilla species from ‘endangered’ to ‘critically endangered’ status in the 2007 Red List of Threatened Species [30], [31].

Infectious diseases could have considerable consequences on genetic characteristics of affected populations [32]. On the one hand, a loss of genetic diversity, an increase of inbreeding and the fixation of deleterious alleles are theoretically expected due to the demographic crash induced by disease [33]–[35]. However, these genetic effects are observed more than 15 generations after a demographic bottleneck [36], which means for long-lived species such as gorillas more than 300 years after a demographic crash. Withal, short term genetic effect of high mortality in a population is the faster reduction of allele numbers than the reduction of gene diversity [37]. The bottleneck effects often go with a decrease in fitness and in disease resistance [38]–[40]. On the other hand, epidemics, because of the intensive selective pressure they exert on populations, play a critical role in population adaptation and evolution [32]. First, epidemic will select for individuals that are genetically the most resistant to the pathogens, i.e. the individuals having the advantageous alleles in case of directional selection or the individuals that are the most genetically diverse in case of balancing selection [41]. Indeed several studies showed that inbred individuals are more prone to deleterious effect of infectious diseases than outbred individuals [42], [43] and some resistant haplotypes could be identified [44], [45]. Other studies bore the evidence that pathogens induced high polymorphism of loci involved in resistance [46], [47]. Secondly, disease selective pressure will change the allele frequencies of unselected loci genetically linked to loci involved in immune response (genetic hitch-hiking [48]).

Besides, high mortality induced by disease in socially structured population could perturb the mating relationships between individuals especially if dominant breeders died. Moreover, after outbreak, vacated territories could be colonized by immigrants, impacting the genetic diversity of the population.

However, most of the studies assessing the impact of disease on genetic diversity and structure of affected populations are conducted without sampling before outbreak and are based on comparisons with unaffected populations or sampling in museums [49]. The studies comparing samples before and after disease outbreaks in the same population are very rare [49], [50].

**Figure 1:**
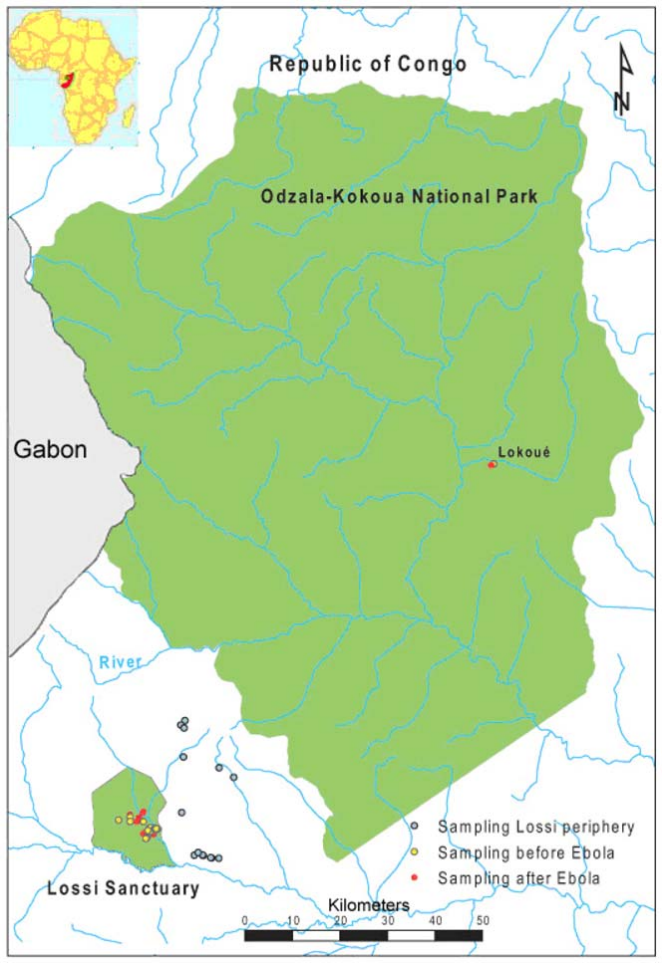
Map of the studied populations. The locations of the samples collected before and after Ebola outbreaks are showed.

We investigated the short term effects of Ebola outbreaks on genetic diversity and structure of the Western lowland gorilla population in the Odzala-Kokoua National Park and in the Lossi Sanctuary (Republic of the Congo, Figure 1). Thanks to the long term monitoring of theses populations, faecal samples were collected from wild gorillas before and 2 to 4 years after Ebola outbreaks and at the periphery of the sanctuary where Ebola has not been detected. 177 different individuals were genetically identified using 17 microsatellite loci. Due to the short time span between sampling events, genetic diversity is expected to be similar in pre and post-epidemic samples assuming that there is no bias in the sampling or in mortality due to Ebola and that social organisation and migration fluxes were not perturbed by the outbreaks. In order to test these hypotheses, we examined temporal changes in genetic diversity and structure in pre and post-epidemic populations. Given that gorilla share Ebola virus with humans and that viability of gorilla is threatened by this disease [2], [51], understanding the influence of infectious diseases on gorilla genetic dynamics is of paramount importance for human health, evolution and conservation biology sciences.

## Materials and Methods

### Studied Populations

Faecal samples were collected in two populations of Western lowland gorillas, *Gorilla gorilla gorilla* in the Republic of the Congo before and after Ebola outbreaks and in one closed population where Ebola has not been detected (Figure 1). The two populations affected by Ebola are in protected areas where deforestation and hunting are restricted. The first population is in the Odzala-Kokoua National Park, which spreads over 13,200 km^2^ and shelters one of the biggest populations of gorilla (averaging density 5.4 ind/km^2^ [52]). The gorillas were monitored since 2001 from a platform placed at the edge of a swampy clearing close to the Lokoué river (0°54.38N, 15°10.55E, Lokoué population [53]). This swampy clearing is very attractive for gorillas that come to feed on herbaceous vegetation, and observational data on individuals were intensively collected using powerful spotting scopes, binocular and a video camera [28], [53], [54]. In 2001–2002, 377 individuals (45 groups and 31 solitary males) were identified [53], [54]. Identified groups were tracked in the forest when they left the clearing to their nest site for the night. Faecal samples were collected along the track or on the nest site when the gorillas were gone, no more than 3 km around the clearing. Ebola outbreak occurred in this population in 2004 inducing high mortality rate (95% [28]). About 400 samples were collected before Ebola outbreak and about 80 samples after. Time span between samples was on average 3 years. The second site is 100 km far away from the Lokoué clearing in the Lossi Sanctuary (Lossi population), a protected area of 320 km^2^. Since 1995, gorillas were habituated to the presence of the Barcelona university staff and by 2002 they had identified 10 social groups with 143 individuals [13], [55], [56]. Gorillas were monitored during their daily movements as far as their nocturnal nest site. Faecal samples were collected in the morning when the gorillas left the site. Ebola outbreak occurred first from October 2002 to January 2003 killing 91% of the individually known gorillas, and then a second time from October 2003 to January 2004, killing 95.8% of individuals in the newly monitored groups [13]. As almost all the individuals have disappeared because of Ebola, sampling in the same area before and after outbreaks was very difficult. About 75 faeces samples were collected before the Ebola outbreak whereas only 21 faecal samples could be collected after Ebola. The time span between the two sampling was about 4.5 years. Therefore investigations at the periphery of the sanctuary, where no carcass infected by Ebola was detected, were performed to monitor and sample the individuals in this area (Figure 1). About 60 faeces samples were collected at the periphery of the sanctuary. In both populations, people who collected the samples wore mask and gloves and used sterile tools in order to avoid contamination. GPS coordinates were noted for each faecal sample. Thanks to observations, faecal samples were attributed to known social groups or known solitary individuals (Table 1), differences between pre and post-epidemic samples in proportion of solitary individuals *vs.* individuals in social group were tested with Fisher's exact tests. Size of the faeces and observations sometimes allowed to determine the age class of the individual. In all sampling all the age classes (juvenile, sub-adult and adult) were represented in an unknown proportion. Samples were dried with silica beads and then stocked at 4°C in the lab before extraction.

**Table 1:** Comparison of group structure and measures of genetic diversity between pre and post-epidemic samples of the Lossi (15 loci) and the Lokoué (17 loci) populations.

	*N*	*Sex ratio*	*Social composition*		*He*	*Ho*	*H_I_*	*A*	*Ar*
			*Social gp.*	*Solitary*					
Lossi:									
Pre-epidemic	68	22/45*	94% (n = 5)	3%	0.752	0.768	0.768	6.69	4.97
Post-epidemic	13	6/5	38% (n = 2)	46%	0.726	0.765	0.756	6.38	5.60
Statistical test		p = 0.0004 †		p<0.001 *^F^*	*p* = 0.25 *^W^*	*p* = 0.9 *^W^*	p = 0.2 †		*p* = 0.13 *^W^*
Lokoué									
Pre-epidemic	42	17/22	100% (n = 27)	0%	0.764	0.759	0.783	7.82	5.25
Post-epidemic	33	17/16	67% (n = 8)	33%	0.758	0.777	0.766	7.41	5.08
Statistical test		p = 0.32 †		p<0.001 *^F^*	*p* = 0.13 *^W^*	*p* = 0.34 *^W^*	p = 0.6 †		*p* = 0.09 *^W^*

*N*, sample size with individuals typed at 10 or more loci; Sex ratio: number of males on the number of females, deviation from balanced sex ratio are noted with *; Social composition: percent of individuals living in social groups with the number of represented groups in parentheses *vs.* percent of solitary individuals - sum could differed from 100% when not all the samples were assigned to known individuals; *He*, *Ho* expected and observed heterozygosity rates, *H_I_*, average individual heterozygosity, *A*, average number of alleles, *Ar*, allelic richness calculated for a sample of 8 diploids individuals, †: χ^2^ test, *^W^*: Wilcoxon's sign rank test, *^F^:* Fisher exact test.

### Extraction and Genotyping

Genomic DNA was extracted from 10 mg of dried faeces, using the 2CTAB/PCI method that increases the DNA amplification success to 94% for gorilla faecal samples [57]. Sex of individuals was determined by amplification of the amelogenin gene (method described in [57]), which allowed us to know the sex-ratio of the samples (Table 1). We tested difference in sex ratio between pre and post epidemic samples using χ^2^ tests. 50, 65, 68, 21, 48 samples, for Lokoué pre-epidemic, Lokoué post-epidemic, Lossi pre-epidemic, Lossi post-epidemic and Lossi periphery population respectively, were genotyped at 17 microsatellite loci that have been characterized for human first. For the Lokoué population before Ebola outbreak, 400 samples were previously genotyped at 6 or 8 microsatellite loci by S. Gatti. We estimated the sample size for which allele frequencies are representative of the population by simulating population with EASYPOP [58]. We used the mean heterozygoty calculated of genotyped loci and fixed the mutation rate to 10^−3^. We found that with 50 individuals observed allele frequencies differed only by 0.05% (SD =  0.00015) from population allele frequencies. We then chose 25 females and 25 males in the sample of 400 individuals to complete their genotypes to 17 microsatellite loci. The 17 microsatellite loci were used because of their high polymorphisms previously detected in gorilla and other primate species (D1s533, D1s548, D1s550, D2s1326, D2s1329, D2s1368, D4s243, D5s820, D5s1470, D6s474, D7s794, D7s817, D10s1432, D16s2624, D18s536, D20s206, vWF, [59]–[63]). PCR mixtures (10 µl final volumes) contained 1 µl of template DNA, 0.16 mM dNTP, 0.4 µM R-Primer, 0.4 µM F-Primer fluorescently labelled with one of 6-FAM (Sigma), VIC, NED, PET (Applied Biosystems), 0.6 U Taq DNA polymerase and 1·X buffer (Qiagen). Cycling was performed under the following conditions: 94°C for 15 min, 50 cycles of 30 s at 94°C, 30 s at 50–60°C, and 30 s at 72°C and a final step of 10 min at 72°C; annealing temperatures depended on loci. PCR products were resolved on a 3130 X DNA sequencer and analyzed using GENESCAN software (Applied Biosystems).

In order to avoid cross contaminations, extraction and pre and post-amplification procedures were performed in different rooms and we used negative controls for each batch of extraction (30 samples), and in all amplifications. A positive control (human) for amplification allowed scaling genotypes between runs. Each allele scored at a heterozygous locus was observed from three or more independent amplifications, while a genotype at a locus was considered homozygous only after scoring five amplifications [64]. Only individuals with complete genotypes at 10 or more loci were included in the analyses.

### Statistical Analysis

#### Marker polymorphism

We used Genecap software [65] to match samples with identical genotypes. When identical genotypes occurred, we kept the most complete one for the analyses. We assessed the reliability of the marker used to study genetic diversity and structure of the samples using the following methods. We calculated false allele rate as the ratio of the number of amplifications with false alleles on the number of positive amplifications and the allelic dropout rate per loci (ADO [66]) as the ratio of the number of amplifications of only one allele on the number of positive amplification for a heterozygous individual. We also used Micro-Checker [67] to detect allelic dropouts. For each locus, we estimated the number of amplification repetitions necessary to have reliable homozygous genotype using the formula *p* = 1−(ADO)*^n^* where *p* is the probability to have a reliable genotype fixed to 0.99, ADO the allele dropout rate and *n* the number of repetitions. GENEPOP v3.4 [68] was used to detect departure from Hardy Weinberg equilibrium at each locus, loci deviating from the equilibrium were excluded from analyses. We also used Fisher's exact tests implemented in GENEPOP v3.4 to test the populations for linkage disequilibrium between all pairs of loci.

#### Assessing the impact of mortality events on genetic diversity

Due to the short time span between pre and post-epidemic samples in both populations, we expected that genetic diversity would be similar in both periods but we suspected a potential reduction in allele numbers. In order to compare them, we calculated mean number of alleles, observed (*Ho*) and expected (*He*) heterozygosity rates for each locus and the mean of this value for each population using GENETIX [69] and allelic richness using FSTAT v2.9.3 [70]. Average individual heterozygosity was estimated by the ratio of the number of heterozygote loci observed to the total number of loci. Comparisons of pre and post-epidemic genetic diversity indexes were performed with relevant non parametric tests (Wilcoxon's signed rank tests for the expected and observed heterozygosity and the allelic richness; Kruskall Wallis test for the average individual heterozygosity). Reduction in rare allele numbers was tested using a qualitative descriptor of allele frequency distribution implemented in BOTTLENECK [71]. L-shaped distribution of allele frequency is expected for stable populations whereas the loss of rare alleles in bottlenecked population should shift the mode toward high allele frequency.

In order to test if the patterns of comparisons between pre and post-epidemic genetic diversity were in accordance with what was expected for populations which experienced high mortality events in a short time, we simulated bottlenecked populations for long lived species with overlapping generations (BOTTLESIM program [72]). We followed the scenario described by Bermejo et al. [13] to simulate two successive high mortality events due to Ebola disease (90% of mortality each time). We simulated four years: at *t* = 0, population had the initial population size (*N_0_* = 5,000; 2,500; 1,000; 500), at *t* = 1, the initial population size was reduced by 90% (first mortality event), at *t* = 2, the population size was stable (*N_2_* = *N_1_*), at *t* = 3, the population size was again reduced by 90% (second mortality event), at *t* = 4, the population size was stable (*N_4_* = *N_3_*). The program assumed that mortality rates were constant among individuals, whereas it had been shown that Ebola affected more female and young individuals in social groups than solitary males [28]. We could not change the proportion of individuals per age class but we could change the proportion of female each year. We first run simulations assuming balanced sex ratio each year, and two other batches of simulation assuming a male biased sex ratio after the bottleneck (1∶2 and 1∶4). As the pattern was similar when high mortality induced sex biased populations or not (Wilcoxon's signed rank test, *p*>0.05), we presented only those results when sex ratio is unbalanced. We chose the dioecious reproductive system with a single reproductive male each year [73], [74]. We defined 100% degree of generation overlapping as all age classes could be observed at the same time in one population [55], [74]. The expected longevity of the organism was set to 40 years and age of reproductive maturation to 10 years [75], [76]. Observed number of alleles and allele frequency in the initial population were specified with the pre-epidemic data for both populations. We run 500 iterations and we compared the number of alleles, expected and observed heterozygosity rates between simulated and observed post-epidemic populations with Wilcoxon's sign rank test.

By reducing the population size, mortality due to Ebola could increase inbreeding. To test this hypothesis, we computed matrices of relatedness coefficients between individuals within samples using the pre-epidemic allele frequencies as a reference (Li's relationship coefficient implemented in SPAGeDI 1.2 software [77], [78]). Comparison between pre and post-epidemic sample for each population was achieved by resampling (999 times) randomly the relevant half matrices, calculating the difference between the means of the pre and post epidemic sample and testing if its distribution was centered on 0 (as expected if relatedness did not differ between the two samples, see [79]).

Reduction in population size is also expected to lead to reduction in effective population size. We estimated effective population size (*Ne*) for both populations before and after the Ebola outbreak, using the approximate Bayesian computation implemented in ONeSAMP [80]. The program first calculates eight statistics on genetic diversity from the input data set. Then it creates 50,000 simulated populations, each population has an effective size drawn from a uniform random number between the lower and upper provided prior *Ne*. We tested four prior limits: [2–500], [2–1,000], [4–5,000] knowing that maximal local population size is about 2,500 individuals [81]. For each simulated population, the program builds samples with the same number of individuals and loci than in the input data and calculates their eight diversity statistics. When summary statistics are closed to the ones calculated for the input data, the *Ne* values of the simulated population is accepted. The *Ne* values from accepted simulated populations are used to estimate the effective size of the input population and its 95% credible limits for the posterior distribution. We also used the bias correction method developed by [82] to estimate *Ne* based on linkage disequilibrium. This method is implemented in LDNe [83] and allows using linkage disequilibrium to estimate *Ne* even when sample size is small. The model assumes selective neutrality of unlinked markers and a single, closed population. However, recent population declines are not suspected to seriously affect estimate of *Ne* [84]. Bias in *Ne* estimate might be expected if allele frequencies are close to 0, we thus excluded alleles with frequencies less than 0.05.

#### Assessing changes in allele frequencies

We tested if distribution of allele frequencies differed between samples with first computing pairwise *F_ST_* (according to [85]), and determining their statistical significance by 10,000 permutations with MSA 3.12 [86].

The changes in allele frequencies between pre and post-epidemic samples in both populations were then computed using an exact homogeneity test implemented in GENEPOP v3.4. However, this test assumes that the samples are independent which is not appropriate when comparing temporally spaced samples of the same population. Therefore, if allele frequencies were significantly different between pre and post-epidemic, we used a generalized test developed by Waples [87] in which the null hypothesis considers that observed difference in allele frequencies are due entirely to stochastic process (i.e. genetic drift or sampling error) in a finite population. Analyses were conducted following sampling plan II (sampled individuals are not replaced before reproduction to mimic the effect of mortality). The temporal Waples' test requires some assumptions about the effective population size (*Ne*), and the number of generation between two samples (*t*). According to confidence interval estimated with both the Bayesian method and the linkage disequilibrium method, *Ne* ranged from 10 to 80 for the Lossi population and 10 to 1,000 for the Lokoué population. Generation time of gorilla is high (estimated to 22 years, D. Caillaud, unpublished data) and lag time between pre and post-epidemic sampling is far from one generation time (about 4 years). Tests were thus conducted with *t* = 1 and *t* = 1/5, using Delphi v.6. The differences in allele frequencies between pre-epidemic and periphery samples and between pots-epidemic and periphery samples in Lossi were computed using only the exact homogeneity tests (GENEPOP v3.4).

Changes in allele proportions could be due to immigration of individuals from a genetically distinct population, selection of individuals carrying genetic variants which influenced their survival, or bias due to low sample size after epizooty. Those hypotheses were hereafter considered.

#### Testing for immigration effect

Presence of first generation migrants in post-epidemic samples was investigated using the program GeneClass2 [88]. The Bayesian statistical approach of [89] was chosen, because it has proven to be more accurate than frequency and distance based methods [90]. As some source populations are clearly missing, the likelihood of the individual genotype within the population where the individual has been sampled, *Lhome*, was used as the test statistic for the detection of first-generation migrants. Low value of *Lhome* indicates that the individual is likely to be a first-generation migrant. We computed for individuals sampled after the epizooties their *Lhome* within the post-epidemic sample and within the pre-epidemic sample. We then compared the distribution of *Lhome* frequencies within pre-epidemic sample and within post-epidemic sample with a *χ^2^* test, in order to test if distribution of *Lhome* within pre-epidemic was skewed toward low value of *Lhome*. For the Lossi population we also computed for individuals sampled after the epizooties their *Lhome* within the periphery sample and tested if the distribution differed from within post-epidemic sample in order to detect if migrants are likely coming from the periphery.

#### Testing for selection pressure

Selection pressure was examined with the test of neutrality of Ewens-Watterson [91], [92]. The test is based on the calculation of the difference (*DH*) between the observed gene diversity (equivalent to the Hardy-Weinberg expected heterozygosity) and the expected heterozygosity under mutation-drift equilibrium with an infinite allele mutation model. This test is thus not based on the comparison before and after the disease. Each *DH* value is divided by the standard deviation (*sd*) of the gene diversity to standardize sample size differences between loci (*DH*/sd) and a significance value is assigned to *DH*/sd using simulations [37]. Negative value of *DH*/sd indicates positive selection due to a high frequency of the selected allele. On the other hand, positive value of *DH*/sd is the sign of balancing selection where polymorphism is maintained. Significance of the neutrality test was evaluated by simulations implemented in BOTTLENECK [71] performed under the two-phase mutation model (TPM) with one-step mutations occurring at a frequency of 95% of the total. This model is considered a more realistic microsatellite mutation model than the infinite allele model [93]. The results with the infinite allele model (IAM) and the single step model (SSM) were also computed for comparison.

#### Testing for sample size bias

Tests of detection of changes in alleles frequencies and of selection pressure could be biased because of the low sample size after Ebola outbreak especially in Lossi. In order to test this hypothesis, we randomly sampled 13 individuals from the Lossi's pre-epidemic population 100 times. We checked that all 100 sub-samples were different. For each sub-sample and each locus, we tested deviation from Hardy Weinberg equilibrium, variation in allele frequencies with exact homogeneity tests and selection using Ewen-Watterson neutrality tests as detailed before. We computed the number of significant tests per locus. We also calculated the mean difference between allele frequencies and its 95% confidence interval in order to test if observed deviations in post-epidemic sample were more or less extreme than within the sub-samples.

## Results

A total of 177 Western lowland gorillas were typed at 10 or more loci. Sex ratio did not differ between the pre and post-epidemic samples in Lokoué. By contrast, in this population, the group composition differed between the two samples since only social groups were sampled in the pre-epidemic sample whereas 33% of genotyped individuals in the post-epidemic sample were solitary individuals. In Lossi, the post epidemic sample differed both in sex ratio and group composition from the pre-epidemic sample: the post-epidemic sample contained proportionally more males and more solitary individuals than the pre-epidemic sample (Table 1).

### Marker Polymorphism

All the loci were polymorphic and the number of alleles per locus ranked from 5 to 28. The mean false allele rate was low with low variation among loci (2.5%, se = 1.1%). According to the ADO for each locus, the number of repetitions needed for reliable homozygous genotypes reached five which was consistent with our protocol. Some loci in Lossi post-epidemic and periphery samples were discarded from the analyses including those samples with low amplification success (D1s533 and D2s1326 in Lossi post Ebola and D1s548 and D2s1368 in Lossi's periphery) or deviation from HWE (D1s548 n = 10, *F_IS_* = 0.64, *p*<0.001 and D20s206 n = 13, *F_IS_* = 0.04, *p* = 0.0009 in the Lossi post-epidemic sample). No linkage disequilibrium was detected between loci after Bonferonni correction.

### Impact of Mortality Events on Genetic Diversity

No comparison of measures of genetic diversity between pre and post-epidemic samples was significant for Lokoué and for Lossi (Table 1). No significant mode shift in the allele frequency distribution due to recent bottleneck in the post-epidemic samples was detected (Fisher tests, *p*>0.05).

**Figure 2:**
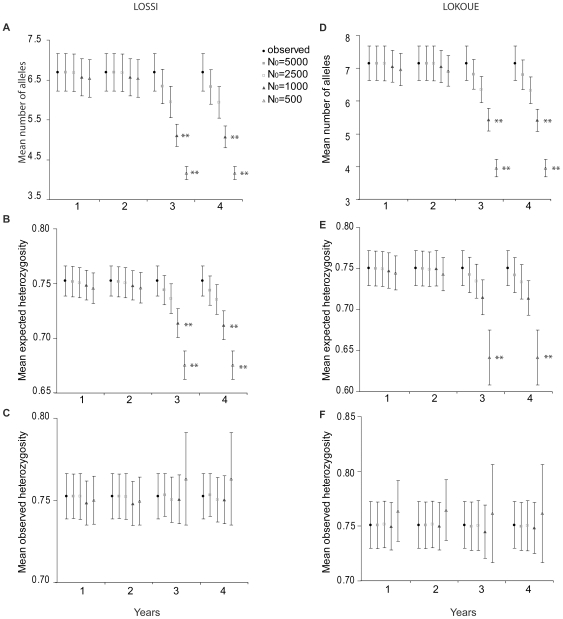
Comparison of genetic diversity indexes between post epidemic and simulated bottlenecked populations. The comparison of number of alleles, expected and observed heterozygosities between post-epidemic population (black circles) and bottlenecked populations simulated for long-lived overlapping generation species with initial population size (*N_0_*) 5,000 (black squares), 2,500 (open squares), 1,000 (black triangles), 500 (open triangles) are presented for Lossi (A, B, C) and Lokoue (D, E, F). Results shown are from simulation with female biased mortality during bottleneck (sex ratio after bottleneck is 1∶2). Allele frequencies of initial population are the ones of pre-epidemic samples of Lossi and Lokoué. Years: 1: just after the first bottleneck (90% of mortality). 2: the population size is constant i.e. equal to year 1. 3: just after the second bottleneck (90% of mortality). 4: the population size is constant i.e. equal to year 3. ** indicates significant Wilcoxon's signed rank test both between observed values and simulated values and between years after the second bottleneck and years after the first bottleneck. Vertical bars represent standard error.

When simulating two successive high mortality events in long lived species with overlapping generations, comparisons of average number of alleles, expected and observed heterozygosities observed in post-epidemic samples and these averages for simulated bottlenecked populations with various initial size (Figure 2) showed that observed averages of diversity indexes were consistent with scenarios of two high mortality events with initial population size of 5,000 and 2,500 individuals for both populations. When the initial population size is 1,000 or 500 individuals, observed averages of diversity indexes were similar to the ones simulated after the first high mortality event. However, the average simulated number of alleles and expected heterozygosity rate after the second high mortality event was significantly lower than observed for both populations (Figure 2, Wilcoxon's signed rank test, *p*<0.05). The average observed heterozygosity was always similar to the simulated observed average.

The mean pairwise relatedness coefficients in pre- and post-epidemic samples did not differ significantly (Lossi *p* = 0.87; Lokoué *p* = 0.45) although its distribution differed significantly between pre and post-epidemic samples in Lossi (Kolmogorov-Smirnov test, *p*<0.0001) but not in Lokoué (Kolmogorov-Smirnov test, *p* = 0.88). Together with a lack of increase of observed heterozygosity (Table 1), these results indicated that Ebola did not increase inbreeding or the risk for inbreeding in these populations.

Estimates of effective size (*Ne*) varied between the linkage disequilibrium and the Bayesian computation methods, and it was also dependent on the prior used for Bayesian computations especially for Lokoué samples (Table 2). In Lossi, after the outbreak, when the sample size was the lowest, all Bayesian estimates were in the same range. Discrepancies between the two methods are not unexpected because they rely on different sets of assumptions. In particular, the linkage disequilibrium method is more restrictive because it assumes selective neutrality, unlinked markers and a closed population, and its sensitivity to population decline has not been tested. Despite these differences, both methods detected a significant decline of *Ne* in Lokoué and in Lossi. Depending on the prior, the Bayesian computation estimated the decrease to reach 70% to 87% in Lossi and 53 to 78% in Lokoué. According to the linkage disequilibrium method, the decrease was 98% in Lokoué and 95% in Lossi.

**Table 2:** Mean effective size estimates and their 95% confidence interval with the corrected linkage disequilibrium method [82] and the Bayesian computation method [80].

	Linkage disequilibrium method	Bayesian computation method		
		Prior distribution		
		[2–500]	[2–1000]	[4–5000]
Lossi pre-epidemic	35.4	56.4	47.6	54.7
	[29.2–43.5]	[49.7–74.3]	[41.6–69.9]	[45.8–94.3]
Lossi post-epidemic	1.9	7.2	8.1	9.2
	[1.5–2.3]	[5.4–9.5]	[6.3–10.4]	[7.1–12.0]
Lokoué pre-epidemic	1,570.4	64.7	138.7	53.9
	[150.4–∞]	[38.3–129.6]	[95.2–237.7]	[34.9–113.4]
Lokoué post-epidemic	32.1	30.2	30.3	12.8
	[23.3–47.9]	[24.6–42.9]	[23.5–44.6]	[8.9–20.8]

Calculations for the linkage disequilibrium method did not use alleles with frequencies less than 0.05.

### Changes in Allele Frequencies

**Figure 3:**
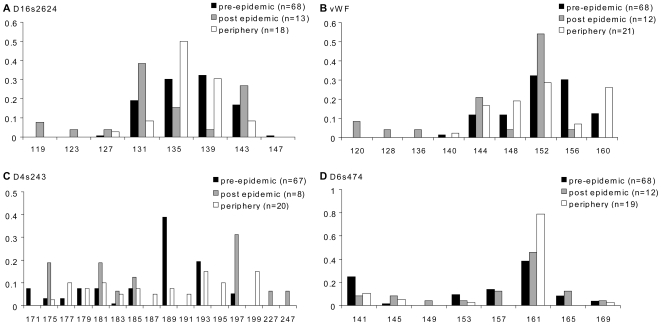
Distribution of allele frequencies of the loci showing significant temporal and spatial changes in Lossi. The pre-epidemic (black bars), post-epidemic (grey bars) and periphery (hatched bars) allele frequencies distribution are presented for the two loci with significant Waples' neutrality tests when comparing pre and post epidemic samples (A and B) and for the two loci with significant homogeneity tests when comparing pre, post epidemic and periphery samples (C and D). n: number of individuals typed for the locus.

Both pairwise *F_ST_* analyses and tests of temporal changes in allele frequencies agreed that no changes occurred in the Lokoué population (Table 3). By contrast, pairwise *F_ST_* between pre, post-epidemic, and periphery samples were significant for the Lossi population (Table 3). Moreover, significant temporal changes in allele frequencies were revealed for four loci (D1s550 n = 12, D4s243 n = 8, D16s2624 n = 13, and vWF n = 12) in Lossi (exact homogeneity tests, Table S1). Expected and observed heterozygoties and mean number of alleles for these loci were all in the 95% confidence interval of the average of these values in the population before Ebola. For these loci Waples' temporal tests were performed to investigate whether stochastic effects alone (sampling error and genetic drift) could explain the heterogeneity in allele frequencies over time. A significant test implies that stochastic effects were not sufficient to explain the differences in allele frequencies. For the Lossi population, the tests were significant for D16s2624 and vWF for all the value of *Ne* tested. The tests for D1s550, and D4s243 loci in Lossi were not significant. Those results indicated that for the Lossi population and the loci D16s2624 and vWF, other factors than stochastic ones were responsible for the observed allele frequency changes between pre and post-epidemic samples (Figure 3A, B).

**Table 3:** *F_ST_* (above diagonal) among samples of Lossi and Lokoué and their statistical significance (calculated with MSA 3.12, below diagonal).

	Lossi pre	Lossi post	Lossi peri	Lokoué pre	Lokoué post
Lossi pre-epidemic	-	0.035*	0.025*	0.016*	0.014*
Lossi post-epidemic	0.001	-	0.034*	0.039*	0.054*
Lossi periphery	0.001	0.003	-	0.020*	0.041*
Lokoué pre-epidemic	0.001	0.001	0.001	-	0.006
Lokoué post-epidemic	0.001	0.001	0.001	n.s.	-

*significant value after Bonferonni correction.

When comparing Lossi sanctuary and periphery, homogeneity tests were significant for three loci (D1s533 n = 17, D4s243 n = 20, and D6s474 n = 19, Table S1, Figure 3B, D) before Ebola and three loci after (D4s243, D16s2624 n =  18and vWF n = 21, Table S1, Figure 3A, B, C).

### Immigration

We found no evidence of immigration. Indeed, the distribution of the likelihood of the post epidemic genotypes (*Lhome*) frequencies did not differ significantly whether it was calculated within the post-epidemic sample or within the pre-epidemic sample for the Lokoué population (*χ^2^*_4_ = 5.9, *p* = 0.21, Figure S1.A) and Lossi population (*χ^2^*_4_ = 0.65, *p* = 0.96, Figure S1.B). Moreover, for Lossi, the distribution of *Lhome* of post-epidemic individuals was significantly skewed toward low values when it was calculated within the periphery sample (*χ^2^*_4_ = 15.03, *p* = 0.004) suggesting that no individuals from the periphery migrated into the sanctuary after the Ebola outbreak. However, two individuals in Lossi had very low *Lhome*, within pre, post-epidemic and periphery samples; those individuals were identified as solitary males.

### Selection Pressure

Significant difference with the neutrality expectation was observed in the post-epidemic Lossi sample for vWF (Ewens Watterson neutrality test, *DH/sd* = −3.14, *p* = 0.01). In the Lossi periphery sample significant differences with the neutrality expectation were observed for D4s243 (*DH/sd* = 1.4, *p* = 0.019) and D6s474 (*DH/sd* = −3.74, *p* = 0.009).

### Sample Size Bias Issue

Analysis of deviation from Hardy Weinberg equilibrium of the 100 sub-samples of 13 individuals from the Lossi pre-epidemic sample showed that only the D1s550 locus deviated from HWE in one sub-sample. Exact homogeneity tests were significant in 1% of the sub-samples for the loci D1s548, D2s1326, D2s1329, D7s817, D10s1432, D16s2624, and D18s536. Neutrality tests revealed that all the loci, except D1s550 and vWF, could show signs of selection in at least 1% of the sub-samples. D16s2624 and D10s1432 deviated significantly from neutrality expectation in respectively 9 and 10% of the sub-samples, indicating potential bias due to sampling for these loci. Observed differences between allele frequencies in post and pre-epidemic samples in Lossi were always superior to the upper limits of the 95% confidence interval of the average difference within the 100 sub-samples.

## Discussion

### Absence of Genetic Diversity Loss

Despite the high mortality induced by Ebola and the significant decline of the effective size estimates, no loss of rare allele was detected after the epizootic. Genetic diversity levels were in the same range than those previously published on Western lowland gorilla [94], [95]. The absence of loss of allelic diversity could be due to immigration in post-epidemic population [96] but the detection of first generation migrant analysis brought no evidence for this hypothesis. Nonetheless, based on the monitoring of identified individuals in Lokoué, four individuals of the post-epidemic population (one silver back, one female, one juvenile and one infant, forming one group) had never been seen in the pre-epidemic population but the likelihood of their genotypes within the post-epidemic population was not different from the mean of the population. Those individuals could originate from a population not genetically differentiated from the Lokoué population, or may had not been detected before the Ebola outbreak. In Lossi, no immigration from sampled periphery had been detected but two individuals, both solitary males, have a low assignment index to all the Lossi samples. They could thus be migrants from a differentiated population that had not been sampled. Despite the suspected low distance dispersal in gorillas [97], [98], previous study on mitochondrial and Y-chromosomal genetic markers [99] showed that some males could travel very long distance during solitary phase. This phenomenon could be enhanced in the context of Ebola outbreaks that decimated whole social groups of related individuals [28]. After the perturbation, the empty territories could be recolonized by individuals or groups from spatially close populations. Social organisation, migration of both sexes and low genetic structure between populations could thus be determinant for the preservation of genetic diversity after the demographic crash.

Another likely explanation for the absence of allelic diversity loss is a high enough remnant effective population size and the short time elapsed after the decline. Indeed, the observed number of allele and expected heterozygosity for both populations were consistent with scenarios of one event of 90% mortality with an initial population size between 500 and 5,000 individuals (remnant populations: 50 to 500 individuals), and scenarios of two events of 90% mortality in high initial population size (remnant population from 25 to 50). Minimum population size and growth rate following the decline are the main factors that influence loss of genetic diversity during high mortality event [100]. The empirical estimates of effective size in each population suggest that this hypothesis is more relevant for Lokoué (*Ne* after epizooties>30) than for Lossi (*Ne* after epizooties <10), although cautions should be made for Lossi's estimates due to the small sample size. The population size of Lokoué gorilla populations after the epizootic would thus have been sufficiently high to retain almost all the genetic diversity, as it has been hypothesized in previous empirical studies on recent demographic crash in other animal populations [49], [101]–[103]. Nevertheless, due to the high mortality induced by Ebola and the potential other threats such as logging and poaching [21], if the gorilla populations do not quickly recover previous epidemic population size, critical threshold of extinction could be reached before we detected its genetic signature.

### Temporal Changes of Allele Frequencies

Heterogeneity in allele frequencies between the pre and the post-epidemic sample was observed only in the Lossi population. The temporal heterogeneity is small for some locus and could be explained by drift or sampling error. By contrast, other factors than drift and sampling errors were responsible for the observed allele frequency changes between pre and post-epidemic samples for two loci (D16s2624 and vWF) of the Lossi population. Genotyping errors could be excluded because of low false allele rates and no detection of allelic dropout. Bias due to low sample size was suspected as deviations from Hardy Weinberg equilibrium have been revealed for two loci. Indeed sample size bias could explain observed deviation form Hardy Weinberg and changes in allele frequencies in Lossi post-epidemic sample for some loci as D1s548 and D16s2624. However for the vWF locus, observed deviations were more extreme than within sub-samples which lead us to investigate other factors responsible for temporal changes. Those factors could be migration, selection or other non random mortality process. Immigration could not be confirmed with the analysis of first generation migrants but, as we mentioned before, some cues indicated that immigration was possible.

The hypothesis that Ebola disease induced a non random mortality through selection in the Lossi population could not be excluded. Indeed, Ewens-Watterson neutrality tests revealed that vWF in the Lossi post-epidemic sample seemed to be under selection as well as two loci (D4s243 and D6s474) in the non affected periphery of Lossi. Selection in the periphery of Lossi is interesting because this population, located between two affected areas, was expected not to be affected by the disease, but no systematic surveys were performed during Ebola outbreaks. Remnant high density of gorillas and chimpanzees [13] agreed with no severe impact of Ebola, if it occurred, in this population. Rejection of neutrality by Ewens-Watterson test may be due to population bottleneck when rare alleles are lost [104]. Nevertheless, the facts that rare alleles were not lost for loci revealing non-neutral pattern (Figure 3) and that not all loci were affected lead us to reject a demographical artefact. Dealing with microsatellite data, the selective pressure hypothesis assumes that loci that show significant temporal changes in allele frequencies are linked to regions involved in immune response. Unfortunately microsatellite linkage map of the gorilla genome is unknown, while similarity with the human one is expected. Interestingly, the locus vWF is the human marker HUMVWFA31 [60], a STR within the gene of von Willebrand factor, which is an adhesive plasma glycoprotein mediating platelet aggregation [105]. Selection pressure of Ebola on genes involved in regulation of coagulation might thus occur although cautions should be taken because of small sample size in post-epidemic sample.

Alternatively, changes in allele frequencies between pre and post-epidemic samples in the Lossi population could stem from other survival bias which led to a bias composition of sampled individuals in the post-epidemic sample. Indeed, previous study, with the data of the Lokoué population monitoring, showed that females living in groups were more affected (97% of mortality) than solitary males (77% of mortality) [28]. Moreover whole social groups, in which relatedness between individuals could be higher than in the population, are decimated by the disease [28]. Consequently, in regards to kin composition of social groups and to male bias in long distance dispersal [99], if the post-epidemic sample contains a higher proportion of solitary males than in the pre-epidemic sample, changes in allele frequency at some loci are expected. Actually, solitary males could carry alleles which are not frequent among the local females but present in other parts of the population. In fact, analysis of sex-ratio and group composition of Lossi samples revealed that the post-epidemic sample contains a higher proportion of males and of solitary individuals than in the pre-epidemic sample. Therefore, the cost of sociality induced by Ebola and the sex biased dispersal of the species could explain the observed changes in allele frequencies as well as deviation from Hardy Weinberg equilibrium as a result of a Walhund effect for the D20s206 locus. Both genetic and demographic approaches agreed on the importance of social organisation and migration of both sexes in response of populations to demographic crash.

### Contrasted Response to Ebola Outbreaks between Populations

However, if Ebola is inducing non random mortality in the Lossi population, no evidence of temporal change in allele frequency has been detected for the Lokoué population. If selection was the main driving force of temporal variation in Lossi for loci vWF similar pattern is expected in Lokoué too. However, the Lossi population suffered of at least two epizootics (2002–3 and 2003–4) of Ebola whereas the population of Lokoué experienced one in 2004 and such differential selective pressure history could explain the discrepancy [106]. Moreover, Lossi is a smaller and more isolated population than Lokoué. Fragmentation is known to enhance disease emergence and spread by bringing species sharing the same pathogens into closer contact [15]. Greater proximity to human and chimpanzee populations could have enhanced susceptibility of Lossi gorilla's population to the disease perturbation. Besides, Morvan et al. [107] suggested that Ebola is more common in forest peripheries and fragments (such as Lossi population) than in the deep forest (such as Lokoué population). Occurrence of different Ebola virus variants in the Lossi and Lokoué populations might also be possible [108].

An alternative hypothesis is that the contrasted pattern between the two populations could arise because of a different composition of sampled individuals. Indeed in Lokoué, sex ratio of the pre- and post-epidemic samples was similar, unlike in Lossi. Moreover, the random choice of individuals among the 400 available samples for the Lokoué pre-epidemic population allowed to estimate representative allele frequencies with 25 social groups represented (1 to 3 individuals sampled per group). By contrast, in Lossi, before Ebola outbreaks five social groups encompassing 64 individuals (on average 12.8±6.9 individuals per group) were sampled. Indeed, the sampling of faeces on the nest site of habituated groups favoured the sampling of whole social groups often constituted of one male and several females and their descendants [55], [73]. However, the distribution of pairwise relatedness coefficients in Lokoué and Lossi before outbreak did not differed significantly (Kolmogorov-Smirnov test, *p* = 0.09). In both populations, after outbreak, the sample contained more solitary individuals than in the pre-epidemic sample. However, the proportion of sampled individuals from social groups after outbreak was higher in Lokoué than in Lossi (χ^2^_1_ = 5.9, *p* = 0.014). Indeed, the Lossi post-epidemic sample encompassed only two social groups whereas the Lokoué post-epidemic sample gathered individuals from 8 different social groups thanks to the higher remnant population size after outbreak in this population. The impact of the cost of sociality on local population genetic structure is therefore expected to be enhanced in small population.

More knowledge on the ecology and the evolution of the virus and gorilla dispersal behaviour following outbreak is needed to understand the direct (selection) and indirect (immigration) impacts of Ebola disease on genetic dynamics of gorilla populations. This necessitates the long term monitoring of primate populations as this approach has the greatest potential to benefit human health, primate health and conservation, and ecosystem sustainability in the long term [51].

## Supporting Information

Figure S1 Distribution of the frequencies of the likelihood of the individual genotypes of post-epidemic individuals. Distribution of the frequencies of the likelihood of the individual genotypes of post-epidemic individuals (Lhome) within the pre-epidemic and the post-epidemic samples for Lokoué population (A) and within the pre-epidemic, the post-epidemic and the periphery samples for Lossi population (B).
(0.04 MB TIF)

Table S1 Homogeneity tests for temporal changes in allele frequencies between pre and post-epidemic period for Lokoué and among pre, post epidemic and periphery samples for Lossi.
(0.05 MB DOC)
