# Supplementary figures and images for: How Ebola Impacts Genetics of Western Lowland Gorilla Populations
Source: PLoS One. 2009 Dec 18;4(12):e8375. doi: 10.1371/journal.pone.0008375 (PMC2791222; doi:10.1371/journal.pone.0008375)

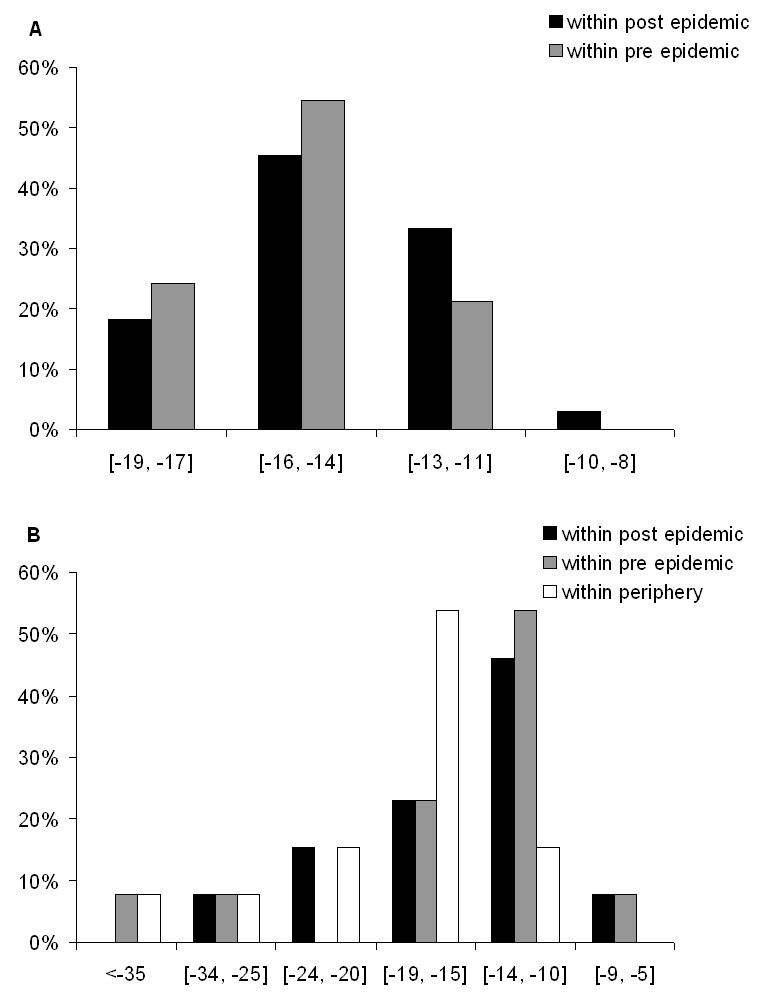

Supplement: Figure S1 — Distribution of the frequencies of the likelihood of the individual genotypes of post-epidemic individuals. Distribution of the frequencies of the likelihood of the individual genotypes of post-epidemic individuals (Lhome) within the pre-epidemic and the post-epidemic samples for Lokoué population (A) and within the pre-epidemic, the post-epidemic and the periphery samples for Lossi population (B). (0.04 MB TIF) [file pone.0008375.s001.tif]
