# Supplementary material for: How Ebola Impacts Genetics of Western Lowland Gorilla Populations
Source: PLoS One. 2009 Dec 18;4(12):e8375. doi: 10.1371/journal.pone.0008375 (PMC2791222; doi:10.1371/journal.pone.0008375)
Supplement: Table S1 — Homogeneity tests for temporal changes in allele frequencies between pre and post-epidemic period for Lokoué and among pre, post epidemic and periphery samples for Lossi. (0.05 MB DOC) [file pone.0008375.s002.doc]

| Locus | Lossi | | | Lokoué |
| --- | --- | --- | --- | --- |
|  | pre/post ebola | pre/periphery | post/periphery | pre/post ebola |
| D1s533 | Not tested | <0.0001* | Not tested | 0.38 |
| D1s548 | 0.075 | Not tested | Not tested | 0.38 |
| D1s550 | <0.0001* | 0.40 | 0.02 | 0.32 |
| D2s1326 | Not tested | 0.14 | Not tested | 0.09 |
| D2s1329 | 0.008 | 0.54 | 0.09 | 0.29 |
| D2s1368 | 0.72 | Not tested | Not tested | 0.04 |
| D4s243 | <0.0001* | <0.0001* | 0.0002* | 0.06 |
| D5s820 | 0.87 | 0.25 | 0.89 | 0.22 |
| D5s1470 | 0.25 | 0.04 | 0.03 | 0.60 |
| D6s474 | 0.07 | <0.0001* | 0.01 | 0.26 |
| D7s794 | 0.25 | 0.01 | 0.40 | 0.61 |
| D7s817 | 0.12 | 0.82 | 0.29 | 0.30 |
| D10s1432 | 0.17 | 0.49 | 0.32 | 0.48 |
| D16s2624 | <0.0001* | 0.13 | <0.0001* | 0.45 |
| D18s536 | 0.01 | 0.86 | 0.28 | 0.012 |
| D20s206 | 0.11 | 0.01 | 0.38 | 0.17 |
| vWF | <0.0001* | 0.01 | 0.002* | 0.15 |
| all loci | <0.0001 † | <0.0001 † | <0.0001 † | 0.01 |

*: significant values after Bonferonni correction, † : significant value p<0.01
